# Subchronic Alcohol Exposure Exacerbates Acetaminophen‐Induced Acute Liver Injury via Macrophage‐Mediated Inflammation and Mitochondrial Dysfunction

**DOI:** 10.1155/mi/4167398

**Published:** 2026-08-17

**Authors:** Qingyang Zhang, Hu Liu, Weigao Zhang, Huiling Zhou, Yunfeng Zhu, Jiayi Xu, Xuening Ma, Kadierya Kuerban, Yong Huang, Lingna Qiu, Fei He, Yiwen Weng, Na Fu, Shao Da, Dan Weng

**Affiliations:** ^1^ School of Environmental and Biological Engineering, Nanjing University of Science and Technology, 200 Xiaolingwei Street, Nanjing 210094, China, njust.edu.cn; ^2^ Department of Emergency Medicine, Nanjing Drum Tower Hospital, The Affiliated Hospital of Nanjing University Medical School, Nanjing, China, nju.edu.cn; ^3^ Internal Medicine Department, Chengdu Jinniu District People’s Hospital, Chengdu, China; ^4^ Department of Traditional and Western Medical Hepatology, Third Hospital of Hebei Medical University, 139 Ziqiang Road, Shijiazhuang, Hebei, 050051, China, hebmu.edu.cn; ^5^ Research Center of Translational Medicine, Shanghai Children’s Hospital, School of Medicine, Shanghai Jiao Tong University, Shanghai 200062, China, sjtu.edu.cn

**Keywords:** acetaminophen, alcohol, inflammation, macrophage, oxidative stress

## Abstract

Alcohol use is a major coexposure in patients taking acetaminophen (APAP), yet how subchronic alcohol exposure reshapes hepatic vulnerability to APAP remains incompletely defined. In this study, we examined the effects of chronic alcohol intake on APAP‐induced liver injury in mice. Our results indicated that subchronic alcohol exposure significantly increased the mortality rate in APAP‐overdosed mice. Alcohol pretreatment also exacerbated APAP‐induced hepatic damage, as evidenced by elevated serum ALT and AST levels, enhanced inflammatory responses, and aggravated oxidative stress. Quantitative polymerase chain reaction (qPCR) analysis revealed that alcohol consumption suppressed mitochondrial biogenesis (MB)‐related genes, including *Pgc-1α* and transcription factor A, mitochondrial (*TFAM*). Notably, macrophage depletion via clodronate liposomes not only attenuated hepatocyte injury and inflammation induction but also restored the expression of MB/function‐related genes, including *Pgc-1α*, *TFAM*, and *Opa1*. These findings suggest macrophage‐involved inflammatory amplification may aggravate APAP‐induced liver injury, at least in part, by impairing mitochondrial adaptive repair responses.

## 1. Introduction

Chronic and excessive alcohol consumption represents a major behavioral risk factor for alcohol‐related harm and imposes a substantial global health burden. According to the World Health Organization, alcohol users accounted for 5.3% of all global deaths in 2018 [1]. In the United States, alcohol‐associated deaths represented 9.8% of total mortality in 2016, with leading causes including cardiovascular disease, alcoholic liver disease (ALD), traumatic injury, and cancer [2, 3].

Alcohol is primarily metabolized in the liver by alcohol dehydrogenase (ADH) and aldehyde dehydrogenase (ALDH), yielding acetaldehyde and acetate. Ethanol and acetaldehyde are also substrates of cytochrome P450 2E1 (CYP2E1), leading to excessive generation of reactive oxygen species (ROS) [3]. Beyond its direct toxicity, chronic alcohol intake enhances susceptibility to other hepatotoxicants, such as acetaminophen (APAP), carbon tetrachloride, nitrosamine, acrylonitrile, and benzene. This is largely attributable to alcohol‐induced upregulation of microsomal and mitochondrial cytochrome P450 enzymes, particularly CYP2E1, which are responsible for metabolizing and activating these compounds, resulting in enhanced metabolism and toxicity [4, 5].

APAP is one of the most widely used analgesics and antipyretics. Although considered safe at therapeutic doses, APAP can cause hepatocyte death in even healthy individuals, and overdose can cause severe hepatotoxicity and even acute liver failure (ALF) [6]. Epidemiological data indicate that over 60 million individuals in the United States use APAP weekly, and APAP‐induced hepatotoxicity has become the leading cause of ALF in the United States and other regions [7–9]. Alarmingly, APAP overdose accounts for ~70,000 hospitalizations and 500 deaths annually in the United States, representing 46% of all ALF cases [7, 10]. Thus, with the widespread usage of APAP, APAP‐induced liver injury remains a major public health issue.

Hepatic APAP metabolism involves glucuronidation and sulfation, which account for 85%–90% of its elimination via urine. About 5%–10% of APAP is metabolized by cytochrome P450 enzymes into the toxic intermediate N‐acetyl‐*p*‐benzoquinone imine (NAPQI) [11]. CYP2E1 and CYP3A4 are the key enzymes responsible for NAPQI production in mice and humans, respectively [12, 13]. NAPQI is normally detoxified by conjugation with glutathione (GSH); however, when NAPQI formation exceeds GSH capacity, it forms covalent adducts with cellular macromolecules, leading to mitochondrial dysfunction, hepatocyte death, and liver injury [14–16].

Although the interaction between alcohol exposure and APAP hepatotoxicity has been investigated for decades, much of the established mechanistic framework has focused on metabolic priming, particularly alcohol‐induced CYP2E1 upregulation, enhanced NAPQI formation, and depletion of hepatic GSH reserves. These mechanisms provide a well‐recognized explanation for the increased susceptibility of alcohol‐exposed livers to APAP overdose. However, APAP‐induced liver injury is not determined solely by the initial bioactivation of APAP. The progression and resolution of injury also depend on mitochondrial stress responses, antioxidant regeneration, hepatocyte death signaling, and innate immune activation. In this context, how chronic alcohol exposure reshapes the downstream inflammatory and mitochondrial adaptive responses after the APAP challenge remains insufficiently defined.

Emerging evidence suggests that macrophages and mitochondrial quality‐control pathways may critically influence the severity of APAP‐induced liver injury, yet their roles in the setting of chronic alcohol exposure remain unclear. In particular, whether subchronic alcohol exposure merely increases APAP toxicity through canonical CYP2E1/GSH‐dependent metabolic mechanisms or additionally establishes a proinflammatory hepatic microenvironment that impairs mitochondrial biogenesis (MB) and amplifies hepatocyte death has not been fully resolved. Therefore, the present study aimed to define the immune–mitochondrial mechanisms by which subchronic alcohol feeding modifies APAP‐induced acute liver injury. Using a chronic 5% ethanol liquid‐diet model followed by APAP overdose, we show that subchronic alcohol exposure aggravates APAP‐induced liver injury in association with enhanced inflammatory signaling and suppressed MB. Importantly, macrophage depletion attenuated liver injury, inflammatory cytokine induction, oxidative stress, and MB defects, suggesting that macrophage‐mediated inflammation is a key amplifier of alcohol‐potentiated APAP hepatotoxicity rather than a mere secondary consequence of hepatocyte necrosis.

## 2. Materials and Methods

### 2.1. Healthy Individuals and Patients Diagnosed With ALD

Liver tissue samples were obtained from patients with ALD who underwent liver biopsy, and the control liver samples were collected from liver transplant donors at the Third Hospital of Hebei Medical University. This study conformed to the ethical standards of the Declaration of Helsinki and was approved by the Ethics Committee of the Third Hospital of Hebei Medical University (Approval Number: W2024‐013‐1). All subjects signed informed consent forms.

### 2.2. Animals

All animal procedures were approved by the Animal Care and Use Committee of Nanjing University of Science and Technology (Approval Number ACUC‐NUST‐20250228011) and were conducted in accordance with institutional guidelines.

All wild‐type C57BL/6J male mice aged 6–8 weeks and weighing 20–22 g were procured from GemPharmatech Co., Ltd. (Nanjing, China). The mice were housed in a temperature‐controlled environment (22 ± 2°C) with free access to water and food under a 12/12‐h light/dark cycle.

For the subchronic alcohol exposure model: Liquid alcohol and liquid control diets were purchased from Trophic Animal Feed High‐tech Co., Ltd., China, and fed according to literature reports [4]. All mice were first fed with a control feed for 5 days to adapt to liquid feed and then switched to an alcohol liquid diet containing 5% alcohol for 10 days, while the control mice continued to consume the control diet. The diet was changed daily, and the weight of mice was recorded regularly.

For the APAP‐induced acute liver injury model: On the evening after changing feed, mice were intraperitoneally injected with 300 mg/kg APAP (TCI, H0190) in phosphate‐buffered saline (PBS) [17, 18]. Control mice were injected with an equal volume of PBS intraperitoneally. The time of death of the mice was recorded.

To clear macrophages, each mouse was intraperitoneally injected with 200 µL clodronate liposomes (Yeasen, 40337ES08) 48 h before APAP injection. Control mice were intraperitoneally injected with 200 µL control liposomes (PBS) (Yeasen, 40338ES08).

Serum and liver samples were collected 15 h after APAP administration. Mice were deeply anesthetized with 3% isoflurane in oxygen using an induction chamber. After the absence of the pedal withdrawal reflex was confirmed, terminal blood samples were collected from the retro‐orbital plexus under deep anesthesia. The mice were subsequently euthanized by cervical dislocation while still under deep anesthesia. Blood samples were allowed to clot at room temperature for at least 30 min and then centrifuged at 5000 rpm for 10 min to obtain the serum. The livers were subsequently collected, sectioned, and either snap‐frozen in liquid nitrogen or fixed in 4% paraformaldehyde for further analysis.

### 2.3. Biochemical Assays

Serum levels of ALT, AST, triglyceride (TG), free fatty acids (FFAs), lactic dehydrogenase (LDH), alcohol and hepatic TG, GSH, and malondialdehyde (MDA) were measured using commercial assay kits following the manufacturer’s instructions. The kits used are listed in Table S1.

### 2.4. Histological Analysis

The liver tissue samples were preserved in a 4% paraformaldehyde solution for at least 24 h before being embedded in paraffin. Subsequently, 4.5‐μm‐thick tissue sections were obtained. Following deparaffinization and rehydration, the tissue sections were stained with hematoxylin and eosin (H&E), subsequently dehydrated, and finally mounted with neutral balsam. The images were captured under a light microscope (MF52‐N; Mshot, Guangzhou, China). The software ImageJ was used to analyze the images.

For neutrophil‐associated infiltration, liver sections were stained with an anti‐MPO antibody using the immunofluorescence procedure described above. MPO‐positive cells were quantified using ImageJ in randomly selected fields from each liver section.

### 2.5. Quantitative Real‐Time Polymerase Chain Reaction (PCR)

Total RNA from mouse liver tissues was extracted using an ultrapure RNA kit (Cwbio, CW0581M) following the manufacturer’s instructions. Briefly, after homogenization in the TRIzon reagent with an ice bath and chloroform extraction, total RNA was adsorbed onto adsorption columns and eluted with RNase‐free water. The concentrations and purity of RNA samples were measured using a NanoDrop spectrophotometer (YSNano‐100; Yeasen Biotech, China). Total RNA was reverse‐transcribed into cDNA using 5× All‐in‐One RT MasterMix (abm, G490). The cDNA samples underwent amplification via PCR on the real‐time PCR system, employing a Hieff UNICON advanced qPCR SYBR Master Mix (Yeasen, 11185ES08). The reference gene used was *Gapdh*. The expression levels of various genes were assessed using the 2^-ΔΔCT^ method. The primer sequences used are detailed in Table S2.

### 2.6. Immunoblotting

Liver tissue samples were homogenized in RIPA lysis buffer (Servicebio, G2002) containing protease inhibitor cocktail (Beyotime, P1005) and phosphatase inhibitor cocktail (Beyotime, P1081) with an ice bath. The homogenates were centrifuged at 15000 rpm for 10 min at 4°C, and the supernatants were collected as protein samples. The concentration of protein samples is quantified by the BCA Protein Quantification Kit (Yeasen, 20201). Then, the protein samples were boiled in 5× SDS‐PAGE sample loading buffer (Beyotime, P0015L) and separated by electrophoresis on 10% SDS‐PAGE gels. The separated proteins were transferred onto 0.2 μm PVDF membranes (Merck Millipore, ISEQ00010) using a transfer buffer. The membranes were then blocked in 5% nonfat powdered milk (w/v) in tris‐buffered saline with 1% Tween‐20 (TBST) and incubated with primary antibodies at 4°C overnight, followed by secondary antibodies for 1 h at room temperature. Finally, the membranes were washed with TBST three times and then developed by an enhanced chemiluminescence (ECL) kit (Beyotime, P0018AS). The images were captured, and quantitative analysis was performed using the software ImageJ. The list of antibodies used is provided in Table S3.

### 2.7. Immunofluorescence and Immunohistochemistry

Immunofluorescence: Following heating at 65°C for 30 min, liver tissue paraffin sections were sequentially immersed in xylene, pure ethanol, and distilled water for deparaffinization and rehydration. Then, sections were soaked in a citric acid antigen repair solution (pH 6.0) (Servicebio, G1202) and heated in a microwave oven to conduct antigen retrieval. After natural cooling, the sections were blocked with 3% bovine serum albumin (BSA) in PBS for 30 min and incubated with primary antibodies at 4°C overnight, followed by secondary antibodies for 1 h at room temperature. Finally, the cell nucleus was counterstained using DAPI (Beyotime, C1006), and the sections were sealed with antifade mounting medium (Beyotime, P0126). For immunohistochemistry, sections were additionally processed with 3% H_2_O_2_ before blocking and developed using DAB after being incubated in secondary antibodies. Finally, sections were stained with the nucleus with hematoxylin and mounted with neutral balsam. The images were captured using an inverted fluorescence microscope (MF52‐N; Mshot, Guangzhou, China).

### 2.8. Triphosphate Nick‐End Labeling (TUNEL) Assay

A terminal deoxynucleotidyl transferase‐mediated deoxyuridine TUNEL assay was performed with the TUNEL brightRed apoptosis detection kit (Vazyme, A113) according to the manufacturer’s instructions. Images were obtained by a fluorescence microscope (MF52‐N; Mshot, Guangzhou, China).

### 2.9. Liver Tissue Transcriptome Sequencing

For the transcriptome analysis, four groups of mouse liver samples were selected: ND group (ND), AD group (AD), ND + APAP group (NA), and AD + APAP group (AA). After euthanizing mice, ~100 mg of liver tissue was divided, frozen in liquid nitrogen, and stored at −80°C for mRNA extraction, library construction, sequencing, and alignment. mRNA extraction, library construction, sequencing, and alignment were performed by Genergy Biotechnology Co. Ltd. (Shanghai, China).

Total RNA was isolated from each liver sample using the RNAmini kit (Qiagen, Germany). RNA quality was examined by gel electrophoresis and with a Qubit (Thermo, Waltham, MA, USA). Strand‐specific libraries were constructed using the TruSeq RNA sample preparation kit (Illumina, San Diego, CA, USA), and 2× 150 bp double‐end sequencing was performed using the Illumina Novaseq 6000 instrument. Skewer software (v0.2.2) was used to remove adapter sequence fragments and low‐quality reads, and data quality was checked by FastQC v0.11.2.

The resulting sequences obtained from the sequencing process were aligned with mouse reference genome GRCm39 using the Spliced Transcripts Alignment to a Reference (STAR, v2.5.2b) software. Software, StringTie (v1.3.1c), was employed to assemble transcripts from the alignment results, thereby generating a gene expression matrix.

### 2.10. Differential Expression and Pathway Enrichment Analysis

The R package “DESeq2” (Version 1.48.1) [19] was used to standardize the gene expression matrix and identify differentially expressed genes (DEGs) with the following thresholds: | log_2_ fold change| > 1 and FDR‐adjusted *p*‐values (*p*.adjust) < 0.05.

Gene set enrichment analysis (GSEA) [20] was performed using the “GSEABase” package (Version 1.70.0) in RStudio (R Version 4.5.0). DEGs were sorted according to the magnitude of log_2_ (fold change) values, and the reference gene set was MH: hallmark gene sets (https://www.gsea-msigdb.org/). Kyoto Encyclopedia of Genes and Genomes (KEGG) pathway enrichment analysis was performed using the “clusterProfiler” package (Version 4.16.0) [21]. The enrichment analysis results were visualized using the “ggplot2” package.

### 2.11. Statistical Analysis

All in vivo experiments were meticulously replicated a minimum of three times to ensure robustness and reliability. The data were presented as means ± standard errors of means (SEMs). Statistical analysis was conducted employing GraphPad Prism 9.5.1 software. Statistical significance was assessed using the *t*‐test or two‐way ANOVA. When *p* < 0.05, it is considered to have a significant difference.

## 3. Results

### 3.1. Subchronic Alcohol Exposure Exacerbates APAP‐Induced Acute Liver Injury in Mice

**Figure 1:**
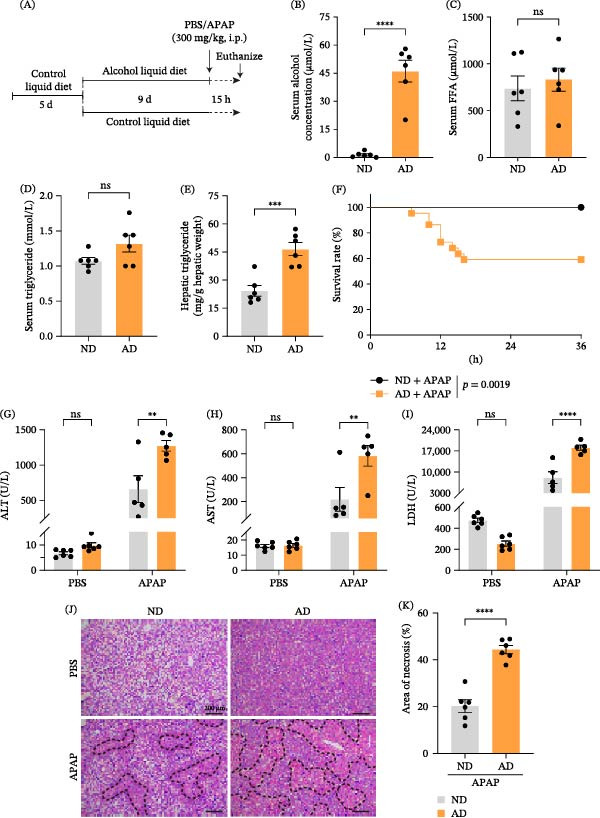
Subchronic alcohol exposure exacerbated APAP‐induced acute liver injury in mice. (A) Experimental procedure. After feeding with a control liquid diet for 5 days, the mice were further divided into groups and fed with a control liquid diet or alcohol liquid diet for 10 days to establish a subchronic alcohol exposure model. The mice were injected with 300 mg/kg APAP intraperitoneally or an equal volume of PBS to establish an acute liver injury model and then were euthanized 15 h after injection. Survival was monitored after APAP administration. Based on the survival kinetics, serum and liver samples were collected at 15 h after APAP injection, a premortality endpoint selected to evaluate liver injury before overt death occurred. (B–D) Serum levels of alcohol, free fatty acids (FFAs), and triglyceride (TG). (E) Hepatic TG content. (F) Survival ratio of mice after APAP administration (300 mg/kg, i.p.). (G–I) Serum levels of ALT, AST, and LDH. (J, K) Representative images and quantitative analysis of HE staining in mouse liver sections. The data were expressed as mean ± SEM and analyzed via two‐way ANOVA or *t*‐test. ns, no significant,  ^∗∗^ *p* < 0.01,  ^∗∗∗^ *p* < 0.001,  ^∗∗∗∗^ *p* < 0.0001.

To investigate the impact of subchronic alcohol exposure on APAP‐induced hepatotoxicity, mice were fed with a 5% ethanol Lieber‐DeCarli liquid diet for 10 days (the NIAAA model) (Figure 1A). Serum ethanol measurements confirmed significantly elevated blood alcohol concentrations in alcohol‐fed mice compared to those in the controls (Figure 1B). Since subchronic alcohol exposure is strongly associated with fatty liver occurrence [22], we also detected serum TG and FFA and hepatic TG levels. Results showed that an alcohol liquid diet significantly increased hepatic TG content but did not markedly alter serum TG or FFA (Figure 1C–E).

Mice were then administered with 300 mg/kg APAP intraperitoneally (Figure 1A). Survival monitoring revealed that in contrast to normal diet‐fed mice, alcohol‐fed mice had a significantly increased mortality rate (40%) following the APAP challenge (Figure 1F). Based on the survival kinetics, 15 h after APAP injection was selected as a premortality endpoint for biochemical and histological analyses, allowing assessment of liver injury before overt death occurred. At this time point, serum and liver samples were collected. Although subchronic alcohol feeding alone had little effect on serum ALT, AST, and LDH, it markedly exacerbated the APAP‐induced elevation of these hepatocellular injury markers (Figure 1G–I). Histological examination with H&E staining confirmed that alcohol feeding significantly aggravated APAP‐induced centrilobular necrosis (Figure 1J,K). Taken together, these results indicated that subchronic alcohol feeding significantly aggravated APAP‐induced mortality and liver injury in mice.

### 3.2. Subchronic Alcohol Exposure Exacerbates APAP‐Induced Oxidative Stress and Mitochondrial Damage in Mouse Liver

**Figure 2:**
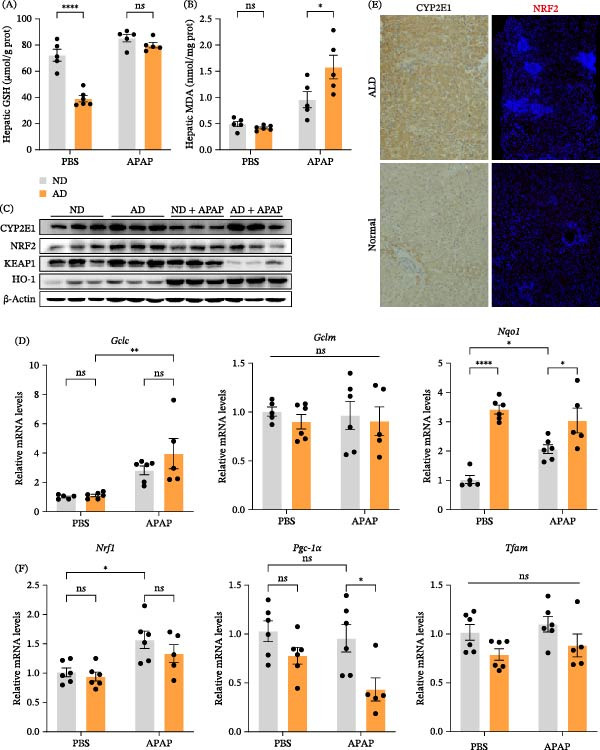
Subchronic alcohol exposure exacerbated APAP‐induced oxidative stress and mitochondrial damage in mouse liver. (A, B) Hepatic GSH and malondialdehyde (MDA) content in mouse liver tissues. (C) Immunoblotting analysis of CYP2E1, NRF2, KEAP1, HO‐1, and β‐actin in mouse liver tissues. (D) The mRNA levels of *Nqo1*, *Gclc*, *Gclm*, *Pgc-1α*, *Nrf1*, and *TFAM* in mouse liver tissues. (E) Representative immunohistochemistry images of CYP2E1 and immunofluorescence images of NRF2 in control donor and ALD patient liver sections. (F) The mRNA levels of *Nrf1*, *Pgc-1α*, and *TFAM* in mouse liver tissues. The data were expressed as mean ± SEM and analyzed via one‐ or two‐way ANOVA. ns, no significant,  ^∗^ *p* < 0.05,  ^∗∗^ *p* < 0.01,  ^∗∗∗∗^ *p* < 0.0001.

GSH is necessary for the degradation of the toxic intermediate NAPQI, and its depletion exacerbates NAPQI‐induced oxidative stress and cell death [23]. Hepatic GSH measurements showed that subchronic alcohol exposure significantly reduced the hepatocellular hepatic GSH reserve (Figure 2A), thereby impairing NAPQI detoxification. MDA, a product of lipid peroxidation and an oxidative stress marker [24], was increased in alcohol‐fed mice after APAP challenge (Figure 2B). Because NAPQI formation is primarily catalyzed by CYP2E1, we assessed its abundance and found that alcohol upregulated CYP2E1 protein levels (Figure 2C). These results indicated that alcohol consumption both upregulated the expression of CYP2E1 and reduced the content of GSH, thereby amplifying APAP‐induced hepatotoxicity.

NF‐E2‐related factor 2 (NRF2) is a key nuclear transcription factor to mediate the expression of many antioxidant genes and coordinate the defense against APAP‐induced oxidative injury. Kelch‐like ECH‐associated protein 1 (KEAP1) can promote the degradation of NRF2, and NRF2 target gene heme oxygenase‐1 (HO‐1) can remove toxic heme to prevent oxidative damage [25] Immunoblotting assay and qPCR analysis revealed no significant differences in the expression levels of NRF2 among groups. Intriguingly, compared with the APAP‐only group, alcohol + APAP reduced the expression of KEAP1 and increased HO‐1 expression (Figure 2C and Figure S1A). Furthermore, we measured the mRNA expression levels of additional NRF2 target genes. We found that alcohol consumption significantly upregulated the mRNA level of NAD(P)H quinone dehydrogenase 1 (*Nqo1*), and alcohol + APAP exhibited a higher *Nqo1* level compared with APAP alone (Figure 2D). To assess the clinical relevance, we examined the expression of CYP2E1 and NRF2 in the liver tissue from patients with ALD. Human ALD liver tissue showed increased CYP2E1 expression and no significant change in NRF2 (Figure 2E).

Mitochondrial quality control (MQC) is an endogenous protective program in cells that maintains mitochondrial homeostasis by coordinating processes such as mitochondrial generation, division, and fusion. Given that APAP metabolism and injury converge on mitochondria, we also detected the expression levels of genes related to MB/functional genes (*Nrf1*, *Opa1*, *Pgc-1α*, and transcription factor A, mitochondrial [*TFAM*]) and fission genes (*Drp1* and *Fis1*) through qPCR (Figure 2F, Figure S1B). Alcohol significantly reduced the expression of MB/function genes (*Pgc-1α*, TFAM) and further suppressed *Pgc-1α* in the presence of APAP.

### 3.3. Bulk RNA Sequencing (RNA‐Seq) Analysis

**Figure 3:**
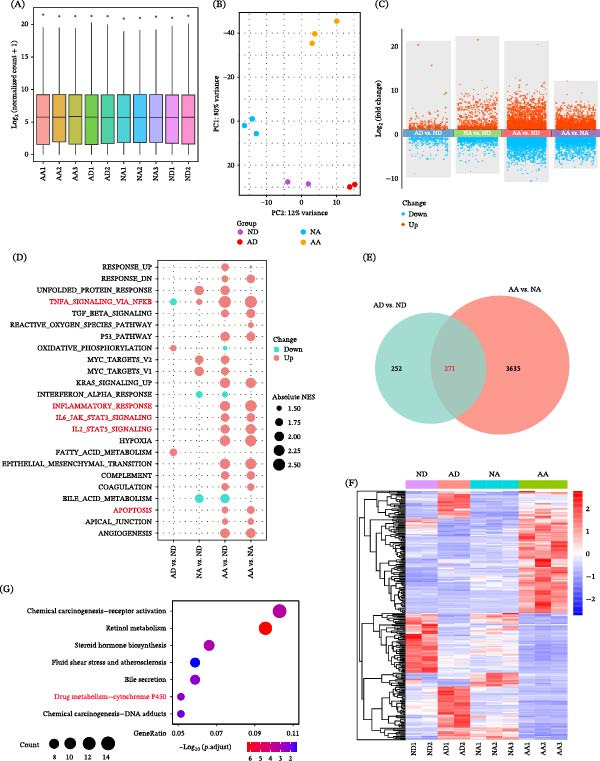
Bulk RNA‐seq analysis. (A) Boxplot of transcript levels in a total of 10 samples. (B) Principal component analysis (PCA) of gene expression in a total of 10 samples of 4 groups: ND, AD, ND + APAP (NA), and AD + APAP (AA). (C) Volcano plot showing up‐ and downregulated genes across 4 groups of differential gene expression analysis. (D) Gene set enrichment analysis (GSEA) showing the enrichment results across 4 groups of differential expression analysis. (E) Venn plot showing the strategy for screening the same DEGs in the liver between AD vs. ND and AA vs. NA. (F) Heat map showing the expression of genes obtained in (E) in all groups. (G) Kyoto Encyclopedia of Genes and Genomes (KEGG) enrichment analysis of the genes obtained in (E).

To further explore the mechanisms by which alcohol‐aggravated APAP‐induced liver injury, we conducted bulk RNA‐seq on mouse liver tissue from the four groups: ND, AD, ND + APAP (NA), and AD + APAP (AA). Following rigorous data preprocessing, we obtained a comprehensive gene expression matrix. Transcript abundance was quantified using normalized counts derived from the R package DESeq2, enabling precise comparison of RNA transcript distributions across samples. Box plot analysis demonstrated consistent median values with minimal intersample variation in normalized count distributions, indicating the absence of significant batch effects (Figure 3A). Subsequently, we used the R package DESeq2 to analyze DEGs on transcriptome data and analyzed the differences between each sample. The plots of principal component analysis (PCA) showed high intragroup consistency and clear intergroup separation (Figure 3B). The volcano plot showed that a substantial number of DEGs were identified in different comparisons (Figure 3C).

Next, GSEA was performed on these DEGs (Figure 3D). Notably, alcohol alone significantly upregulated oxidative phosphorylation and fatty acid metabolism while downregulating TNF signaling via NF‐кB. In contrast, APAP treatment upregulated TNF signaling via NF‐кB and the unfolded protein response and downregulated bile acid metabolism. Most strikingly, alcohol + APAP further enhances TNF signaling, apoptosis, and IL‐6 and IL‐2 signaling compared with the APAP alone group. To identify the key genes of alcohol‐aggravated APAP‐induced liver injury, we intersected the DEGs identified from AD vs. ND and AA vs. NA, and 271 candidate genes were obtained (Figure 3E). Unsupervised clustering showed distinct expression patterns among groups (Figure 3F). KEGG enrichment analysis showed that these genes were enriched in steroid hormone biosynthesis, drug metabolism‐cytochrome P450, and bile secretion pathways (Figure 3G). The enrichment of the cytochrome P450 pathway was consistent with the upregulation of CYP2E1 protein levels in the alcohol‐fed mouse liver.

### 3.4. Subchronic Alcohol Exposure Exacerbates APAP‐Induced Cell Death and Hepatic Inflammation

**Figure 4:**
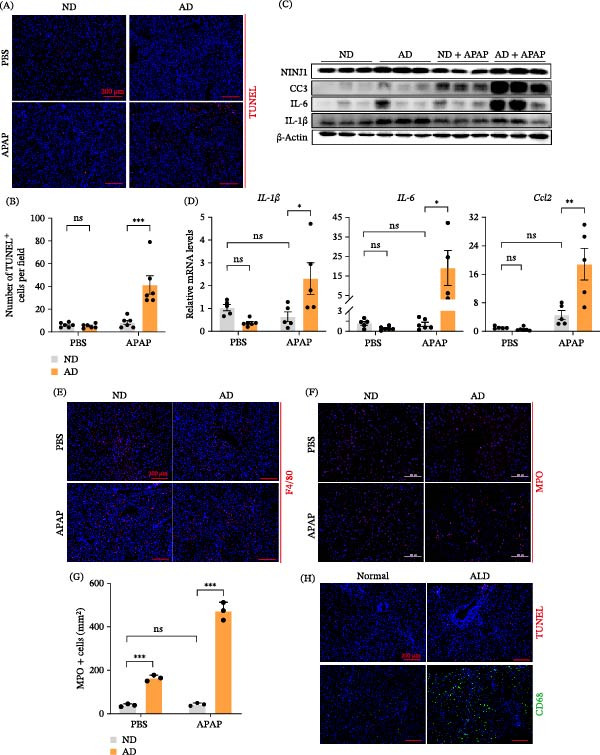
Subchronic alcohol exposure exacerbated APAP‐induced cell death and inflammatory response in mouse livers. (A, B) Representative images and quantitative analysis of TUNEL staining in mouse liver sections. (C) Immunoblotting analysis of NINJ1, cleaved‐caspase 3 (CC3), *IL-6, IL-1β*, and β‐actin in mouse liver tissues. (D) The mRNA levels of *IL-1β*, *IL-6*, and *Ccl2* in mouse liver tissues. (E) Representative immunofluorescence images of F4/80 in mouse liver sections. (F) Representative images and quantitative analysis of MPO immunofluorescence staining in mouse liver sections. (G) Quantification of MPO‐positive cells in staining sections. (H) Representative TUNEL staining images and immunofluorescence images of CD68 in control donor and ALD patient liver sections. The data were expressed as mean ± SEM and analyzed via a two‐way ANOVA. ns, no significant,  ^∗^ *p* < 0.05,  ^∗∗^ *p* < 0.01,  ^∗∗∗∗^ *p* < 0.0001.

APAP overdose is known to induce hepatocellular injury, predominantly through necrotic cell death accompanied by mitochondrial dysfunction and inflammatory activation [23]. Our RNA‐seq analysis also suggested enrichment of cell death‐ and inflammation‐related pathways in alcohol‐fed and APAP‐treated mice. Therefore, we assessed cell death and inflammatory responses in mouse liver tissues (Figure 3D). TUNEL staining and quantification showed that alcohol significantly increased TUNEL‐positive hepatocytes after APAP challenge compared with APAP alone (Figure 4A,B). Immunoblotting analysis confirmed that alcohol and APAP cotreatment increased the levels of cleaved caspase‐3 (CC3) and NINJ1 (Figure 4C), markers associated with cell death. Necrotic cells could release damage‐associated molecular patterns (DAMPs), which activate innate immune pathways [26]. Quantitative PCR (qPCR) analysis demonstrated that the alcohol‐APAP cotreatment significantly upregulated the expression of proinflammatory cytokines (*IL-1β*, *IL-6*) and chemokines (*Ccl2*), whereas APAP alone had a negligible effect (Figure 4D). CCR2, the primary receptor for CCL2, mediates the recruitment of monocytes and macrophages to sites of inflammation. Immunofluorescence staining revealed that APAP treatment increased F4/80+, CCR2+, and CD11b+ cells in the mouse liver. Compared with APAP alone, alcohol pretreatment did not further increase F4/80+ or CCR2+ cells but promoted CD11b+ myeloid cell infiltration (Figure 4E and Figure S1C). Because CD11b is expressed by multiple myeloid populations and cannot distinguish recruited monocytes/macrophages from neutrophils, we further performed MPO staining to evaluate neutrophil‐associated infiltration. MPO‐positive cells were rarely observed in control livers but increased after APAP challenge; this increase was further enhanced in alcohol‐pretreated mice following APAP administration (Figure 4F,G). These results suggest that alcohol pretreatment amplifies APAP‐induced hepatic inflammation through a broader myeloid inflammatory response, involving neutrophil‐associated infiltration in addition to macrophage/monocyte‐related inflammatory signaling. In addition, we examined hepatocellular damage and immune infiltration in the liver tissue from ALD patients. Histological analysis revealed increased TUNEL‐positive cells and CD68^+^ macrophage infiltration in human ALD specimens (Figure 4H). Notably, our alcohol‐feeding regimen alone did not cause overt liver injury, suggesting that alcohol pretreatment amplifies APAP toxicity independent of baseline liver damage.

### 3.5. Macrophage Depletion Alleviates Alcohol and APAP‐Induced Liver Injury in Mice

**Figure 5:**
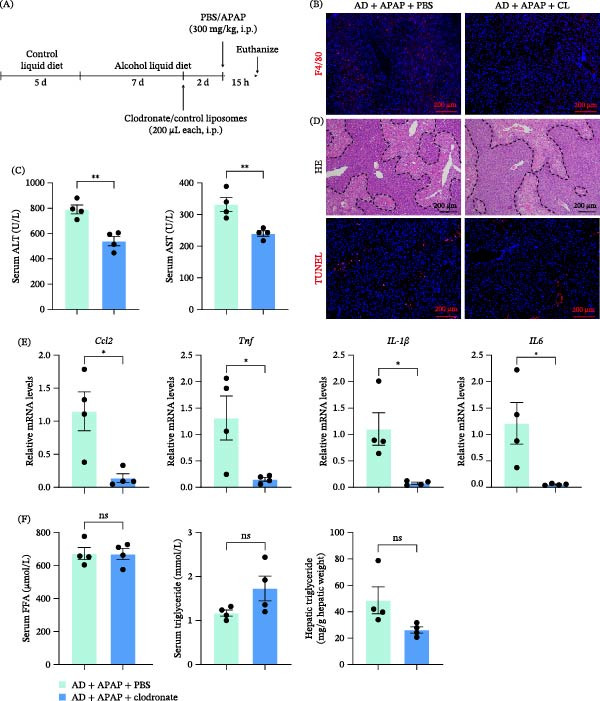
Macrophage depletion alleviated alcohol and APAP‐induced liver injury in mice. (A) Experimental procedure. Mice were fed a liquid alcohol diet as previously described and were injected with clodronate or PBS liposomes (200 μL per mouse, i.p.) 2 days before APAP injection. (300 mg/kg, i.p.). (B) Representative immunofluorescence images of F4/80 in mouse liver sections. (C) Serum levels of ALT and AST. (D) Representative images of HE staining and TUNEL staining in mouse liver sections. (E) The mRNA levels of *Tnf*, *IL-1β*, *IL-6*, and *Ccl2* in mouse liver tissues. (F) Serum levels of FFA and TG, and hepatic TG content. The data were expressed as mean ± SEM and analyzed via *t*‐test. ns, no significant,  ^∗^ *p* < 0.05.

The above results suggest that APAP treatment promoted macrophage recruitment in mouse liver, while alcohol consumption attenuated this effect. In contrast, RNA‐seq and qPCR analysis indicated that alcohol consumption exacerbated the APAP‐induced inflammatory response. Because the alcohol + APAP group exhibited enhanced inflammatory cytokine induction at the 15 h premortality endpoint, we next examined whether macrophages contributed to this late injury‐amplification phase. To this end, we performed macrophage depletion using clodronate liposomes. Following an established mature protocol, mice were fed a liquid alcohol diet and injected with 300 mg/kg APAP; clodronate liposomes were injected intraperitoneally 48 h before APAP administration (Figure 5A). F4/80 staining confirmed that clodronate liposomes successfully depleted macrophages in the mouse liver (Figure 5B). Serum biochemical assays showed that macrophage depletion reduced serum ALT/AST levels (Figure 5C). Histological analysis and TUNEL staining also showed that macrophage depletion markedly suppressed APAP‐induced hepatocyte death (Figure 5D). qPCR analysis demonstrated that macrophage depletion significantly downregulated the expression of proinflammatory cytokines (*Tnf*, *IL-1β*, and *IL-6*) and chemokines (*Ccl2*) in mouse liver (Figure 5E). In addition, we also investigated the potential impact of macrophage depletion on the lipid metabolism. Results demonstrated that macrophage depletion led to a decrease in serum TG levels and an increase in hepatic TG content, while no significant change was observed in serum FFA levels (Figure 5F).

### 3.6. Macrophage Depletion Restores MB‐Related Gene Expression in in Alcohol‐ and APAP‐Treated Mice

**Figure 6:**
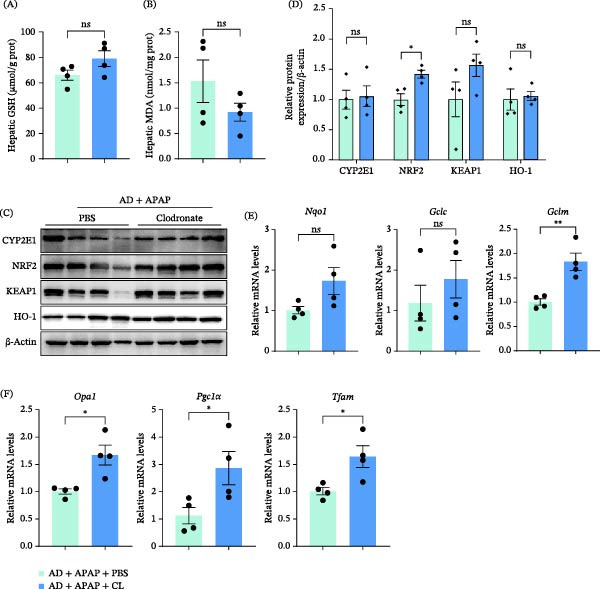
Macrophage depletion alleviated alcohol and APAP‐induced oxidative stress and mitochondrial damage in mouse liver. (A, B) Hepatic GSH and MDA content in mouse liver tissues. (C, D) Immunoblotting analysis and quantification of CYP2E1, NRF2, KEAP1, HO‐1, and β‐actin in mouse liver tissues. (E) The mRNA levels of *Nqo1*, *Gclc*, and *Gclm* in mouse liver tissues. (F) The mRNA levels of *Opa1*, *Pgc-1α*, to analyze images, and *TFAM* in mouse liver tissues. The data were expressed as mean ± SEM and analyzed via *t*‐test. ns, no significant,  ^∗^ *p* < 0.05,  ^∗∗^ *p* < 0.01.

To elucidate the mechanism through which macrophage depletion alleviated alcohol and APAP‐induced liver injury, we measured the levels of GSH and MDA in mouse liver. The results indicated that macrophage depletion increased the GSH content and reduced the MDA content in mouse liver, although not significantly (Figure 6A,B). Immunoblot analysis revealed that macrophage depletion had no effect on CYP2E1 expression (Figure 6C,D), suggesting that it does not influence the metabolism of APAP in the liver. Further analysis of antioxidant pathway‐related proteins and genes showed that macrophage depletion significantly upregulated the expression of NRF2 and its target gene *Gclm* (Figure 6E and Figure S2A). qPCR analysis of MQC‐related genes showed that macrophage depletion significantly upregulated the expression of mitochondrial biogenic/functional genes (*Opa1*, *Pgc-1α*, and *TFAM*) (Figure 6F and Figure S2B). These findings suggest that macrophage depletion attenuates alcohol + APAP‐induced liver injury without reducing CYP2E1 expression by promoting GSH regeneration and enhancing MB and function.

## 4. Discussion

In this study, we established a NIAAA model without a final alcohol gavage and induced liver injury using a moderate dose of APAP. The ethanol liquid diet increased hepatic TG content but did not cause overt liver injury, indicating that this subchronic alcohol exposure model induced early hepatic lipid accumulation without baseline hepatocellular damage [4]. Notably, subchronic alcohol exposure significantly exacerbated APAP‐induced liver injury, as evidenced by increased mortality, elevated serum ALT/AST levels, and aggravated histological necrosis [27, 28]. Intriguingly, after subchronic alcohol exposure, APAP induced a stronger inflammatory response without a corresponding further increase in F4/80+ or CCR2+ macrophage/monocyte‐associated infiltration [29]. To investigate whether macrophage‐involved inflammatory signaling contributes to this injury‐amplification phase, clodronate liposomes were used to deplete hepatic macrophages [30]. Macrophage depletion significantly reduced hepatic inflammatory cytokine expression and alleviated liver injury induced by alcohol and APAP. Together with the newly observed increase in MPO‐positive cells in the alcohol + APAP group, these findings suggest that subchronic alcohol exposure amplifies APAP‐induced liver injury through a broader myeloid inflammatory response, in which macrophage‐involved cytokine signaling and neutrophil‐associated infiltration may both contribute [30].

APAP is metabolized by CYP2E1 into the toxic NAPQI, which is then detoxified by GSH. Therefore, hepatic GSH reserve is crucial for the hepatotoxicity of APAP. In this study, subchronic alcohol exposure upregulated CYP2E1 expression and reduced the hepatic GSH reserve in mouse liver, thereby enhancing APAP hepatotoxicity. These observations align with previous reports that acute alcohol gavage inhibited GSH metabolism at the protein level, thereby reducing the hepatic GSH reserve. These observations align with previous reports that ethanol exposure can impair GSH metabolism and regeneration, thereby aggravating APAP‐induced liver injury [17, 31]. However, we also observed that hepatic GSH levels at 15 h after APAP injection were higher in the alcohol + APAP group than in the APAP‐only group. This apparent paradox is consistent with previous observations that GSH levels may recover or even increase during the late phase of APAP‐induced liver injury [18], likely reflecting compensatory activation of GSH resynthesis in surviving hepatocytes and stress‐adaptive antioxidant responses. Therefore, the GSH value measured at this late premortality endpoint should not be interpreted simply as the initial detoxification capacity during early APAP metabolism [17, 32]. Rather, it may represent a secondary adaptive response to severe oxidative and inflammatory injuries [33]. This finding also suggests that although CYP2E1 induction and basal GSH depletion contribute to the early susceptibility of alcohol‐fed mice to APAP toxicity, the aggravated liver injury observed at 15 h is unlikely to be explained by persistent GSH depletion alone [17, 34]. Instead, downstream inflammatory amplification and impaired mitochondrial adaptive repair may contribute importantly to the progression of alcohol‐potentiated APAP hepatotoxicity [35, 36].

The KEAP1‐NRF2 anti‐oxidation axis plays a critical role in resistance to APAP‐induced liver injury [25, 37, 38]. Typically, KEAP1 binds to NRF2 and promotes its ubiquitin‐mediated degradation. Upon oxidative stress, KEAP1 dissociates from the complex and undergoes degradation, allowing NRF2 to translocate into the nucleus and activate antioxidant gene expression [39]. In our study, cotreatment with alcohol and APAP significantly reduced KEAP1 levels compared to APAP treatment alone, concomitant with elevated MDA levels. These results indicate that subchronic alcohol exposure exacerbates APAP‐induced oxidative stress, potentially through the dysregulation of the KEAP1‐NRF2 pathway.

MQC includes multiple processes such as MB, fission, fusion, and autophagy and is crucial for maintaining mitochondrial homeostasis and function. As mitochondria are major targets of APAP‐induced hepatotoxicity, efficient mitochondrial repair and replacement are essential for limiting oxidative stress and hepatocyte death [40]. Of particular importance is MB, which plays a critical role in APAP‐induced liver injury. *Pgc-1α* is a master regulator that interacts with other transcription factors to induce MB‐related gene expression [41]. Du et al. [42] demonstrated that MB and *Pgc-1α* upregulation selectively occur in the necrotic area of liver cells in APAP‐induced liver injury. The MB inducer SRT1720 increased *Pgc-1α* expression and liver regeneration, thereby alleviating liver damage caused by APAP overdose. TFAM is a key regulator of the replication, transcription, and repair of mitochondrial DNA (mtDNA) [43]. Genetic studies have shown that TFAM heterozygous knockout can induce mitochondrial genome instability and cytosolic mtDNA release [44, 45].

In the present study, subchronic alcohol exposure significantly reduced the expression of MB/function‐related genes, including *Pgc-1α* and *TFAM* [46, 47], suggesting that alcohol exposure may impair adaptive mitochondrial repair after APAP challenge [42, 48]. Importantly, macrophage depletion restored the expression of *Pgc-1α*, TFAM, and Opa1 in alcohol‐ and APAP‐treated mice [49], while also attenuating inflammatory cytokine induction and liver injury [50]. These findings provide a potential mechanistic link between macrophage‐involved inflammatory activation [49, 51] and impaired MB in alcohol‐potentiated APAP hepatotoxicity. Rather than acting solely through the classical CYP2E1/GSH‐dependent metabolic pathway, chronic alcohol exposure may also compromise mitochondrial recovery capacity through inflammatory amplification [27, 52]. However, because we did not directly block specific cytokines such as TNFα or IL‐1β, the causal relationship between inflammatory mediators and suppression of the *PGC-1α*–TFAM axis requires further investigation.

A limitation of the present study is that most mechanistic analyses were performed at a single 15 h time point after APAP administration (Figure 1A). This time point was selected as a premortality endpoint based on the survival curve, allowing us to examine liver injury before overt death occurred. However, this design does not allow us to fully distinguish whether the increased inflammatory response in alcohol + APAP‐treated mice reflects direct priming of Kupffer cells/myeloid cells by repeated alcohol exposure [29, 53, 54] or a secondary response to earlier and more severe hepatocellular injury [55–57]. An earlier time point, such as 6 h after the APAP challenge, would be useful to define the early metabolic and mitochondrial injury phase before robust inflammatory amplification. Our data support the interpretation that macrophage‐involved inflammation contributes to the late injury‐amplification phase of alcohol‐potentiated APAP hepatotoxicity. In addition, although MPO staining supports neutrophil‐associated infiltration in alcohol‐pretreated mice after APAP challenge [58, 59], we did not perform Ly6G‐based neutrophil depletion, lineage‐resolved flow cytometry, or double immunostaining to distinguish neutrophils, recruited monocytes, and resident Kupffer cells. Therefore, the functional contribution of neutrophils to alcohol‐potentiated APAP injury remains to be determined.

In APAP‐induced liver injury, the resident macrophages of the liver (Kupffer cells) recognize DAMPs released from necrotic hepatocytes and secrete the chemokine CCL2, which recruits circulating monocytes to the injury site [23]. In our study, APAP administration without fasting induced liver injury, upregulated CCL2 expression, and increased F4/80+ and CCR2+ cell infiltration but caused only limited induction of proinflammatory cytokines such as IL‐1β and IL‐6 at the 15 h endpoint. Interestingly, although alcohol and APAP cotreatment exacerbated liver damage and increased inflammatory cytokine levels, it did not further enhance F4/80+ or CCR2+ cell infiltration compared with APAP alone. Instead, alcohol pretreatment promoted CD11b+ myeloid cell infiltration and increased MPO‐positive cells after the APAP challenge, indicating that neutrophil‐associated infiltration may be involved in the aggravated inflammatory phenotype. Although recruited neutrophils and monocyte‐derived macrophages typically clear cellular debris to facilitate tissue repair, they may also contribute to further tissue damage [55, 60]. Following cotreatment with alcohol and APAP, the depletion of hepatic macrophages by clodronate liposomes led to downregulation of CCL2, IL‐1β, and IL‐6 expression, accompanied by a partial mitigation of liver injury. However, residual liver injury remained more severe than that induced by APAP alone. These findings suggest that subchronic alcohol exposure exacerbates APAP‐induced liver injury by amplifying macrophage‐mediated inflammatory responses. Together with the restoration of *Pgc-1α*, TFAM, and Opa1 expression after clodronate treatment, these results suggest that macrophage‐involved inflammation may contribute not only to cytokine amplification but also to the suppression of MB/adaptive repair during alcohol‐enhanced APAP liver injury.

## 5. Conclusion

In summary, subchronic alcohol exposure exacerbates APAP‐induced liver injury by upregulating CYP2E1, depleting GSH, impairing MB, and amplifying myeloid cell‐associated inflammatory responses. Notably, MPO staining further suggests neutrophil‐associated infiltration in alcohol‐pretreated mice after APAP challenge, while macrophage depletion alleviates liver injury and restores MB/function‐related genes. Our findings provide novel mechanistic insights into how chronic alcohol intake aggravates APAP hepatotoxicity and may inform clinical strategies for managing APAP overdose in individuals with alcohol use disorder.

## Funding

This work was supported by the National Natural Science Foundation of China (Grants 22376100, 31970897, and 32100828), the Chengdu Medical Research Project (Grant 2024242), and the National Key R&D Program of China (Grant 2024YFF0619500).

## Conflicts of Interest

The authors declare no conflicts of interest.

## Supporting Information

Additional supporting information can be found online in the Supporting Information section.

## Supporting information


**Supporting Information** Appendix A. Supporting information includes the following charts: Table S1: Kits used in this paper. Table S2: Primers used in this paper. Table S3: Antibodies used in this paper. Figure S1: subchronic alcohol exposure exacerbates APAP‐induced oxidative stress and inflammatory response. Figure S2: Macrophage depletion alleviates alcohol and APAP‐induced oxidative stress.

## Data Availability

The data supporting the findings of this study are available within the article and its Supporting Information. Additional processed data and analysis files are available from the corresponding author upon reasonable request. Due to ethical and privacy considerations, individual‐level clinical information related to human liver samples is not publicly available.
