# Supplementary material for: Subchronic Alcohol Exposure Exacerbates Acetaminophen‐Induced Acute Liver Injury via Macrophage‐Mediated Inflammation and Mitochondrial Dysfunction
Source: Mediators Inflamm. 2026 Aug 17;2026:4167398. doi: 10.1155/mi/4167398 (PMC13479109; doi:10.1155/mi/4167398)
Supplement: Supplementary file 1 [file MI-2026-4167398-s001.docx]

**Supplementary Materials for:**

**Chronic alcohol consumption** **exacerbates acetaminophen-induced acute liver injury via** **macrophage-mediated inflammation and mitochondrial dysfunction**

Hu Liu^1^, Weigao Zhang^1^, Huiling Zhou^1^, Yunfeng Zhu^1^, Jiayi Xu^1^, Xuening Ma^1^, Qingyang Zhang^1^, Kadierya Kuerban^1^, Yong Huang^1^, Lingna Qiu^1^, Fei He^2^, YiwenWeng^3^, Na Fu^4, *^, Da Shao^5, *^, Dan Weng^1^

This document includes:

•	Table S1, S2, S3

•	Supplementary figures (Fig. S1, Fig. S2)

**Table S1: Kits used in this paper**

| **Kit** | **Source** | **Identifiers** |
| --- | --- | --- |
| Alanine aminotransferase Assay Kit | Nanjing Jiancheng Bioengineering Institute | Cat#: C009-2-1 |
| Aspartate aminotransferase Assay Kit | Nanjing Jiancheng Bioengineering Institute | Cat#: C010-2-1 |
| Lactate dehydrogenase assay kit | Nanjing Jiancheng Bioengineering Institute | Cat#: A020-2-2 |
| Triglyceride assay kit | Nanjing Jiancheng Bioengineering Institute | Cat#: A110-1-1 |
| Malondialdehyde (MDA) assay kit (TBA method) | Nanjing Jiancheng Bioengineering Institute | Cat#: A003-1-2 |
| Reduced glutathione (GSH) assay kit | Nanjing Jiancheng Bioengineering Institute | Cat#: A006-2-1 |
| Triglyceride (TG) Content Assay Kit | Solaibio | Cat#: BC0625 |
| Free fatty Acids (FFA) Content Assay Kit | Solaibio | Cat#: BC0590 |
| Ethanol Content Assay Kit | Solaibio | Cat#: BC6035 |
| TUNEL BrightRed Apoptosis Detection Kit | Vazyme | Cat#: A113-03 |
| BeyoECL Star | Beyotime | Cat#: P0018AS |
| BCA Protein Quantification Kit | Yeasen | Cat#: 20201ES76 |
| Ultrapure RNA Kit | CWBio | Cat#: CW0581M |
| OneScript® Hot 5X RT MasterMix | abm | Cat#: G590 |

**Table S2: Primers used in this paper**

| **Gene** | **Forward** | **Reverse** |
| --- | --- | --- |
| m-Gapdh | AGGTCGGTGTGAACGGATTTG | TGTAGACCATGTAGTTGAGGTCA |
| m-Nfe2l2 | CAGCATAGAGCAGGACATGGAG | GAACAGCGGTAGTATCAGCCAG |
| m-Keap1 | ATCCAGAGAGGAATGAGTGGCG | TCAACTGGTCCTGCCCATCGTA |
| m-Hmox1 | CACTCTGGAGATGACACCTGAG | GTGTTCCTCTGTCAGCATCACC |
| m-Nqo1 | GCCGAACACAAGAAGCTGGAAG | GGCAAATCCTGCTACGAGCACT |
| m-Gclc | ACACCTGGATGATGCCAACGAG | CCTCCATTGGTCGGAACTCTAC |
| m-Gclm | TCCTGCTGTGTGATGCCACCAG | GCTTCCTGGAAACTTGCCTCAG |
| m-Fis1 | AAGAGGAACAGCGGGACTATG | GGATTTGGACTTGGAGACAGC |
| m-Dnm1l | GCGAACCTTAGAATCTGTGGACC | CAGGCACAAATAAAGCAGGACGG |
| m-Nrf1 | GGCAACAGTAGCCACATTGGCT | GTCTGGATGGTCATTTCACCGC |
| m-Opa1 | AGAATCGGACCCAAGAGCAG | TTCGCCAAAACAGGACCAC |
| m-Pgc-1α | GAATCAAGCCACTACAGACACCG | CATCCCTCTTGAGCCTTTCGTG |
| m-Tfam | GCATCCCCTCGTCTATCAGTC | CCTCCTTCTCCATACCCATCA |
| m-Ccl2 | GCAGGTGTCCCAAAGAAG | ACATTCAAAGGTGCTGAAGA |
| m-Tnf | TCGAGTGACAAGCCTGTAG | GGTGAGGAGCACGTAGT |
| m-Il6 | ACAAGAAAGACAAAGCCAGAG | GTGATTTCAAGATGAATTGGATGG |
| m-Il-1β | GGGCCTCAAAGGAAAGAAT | GGTGCTGATGTACCAGTTG |

**Table S3: Antibodies used in this paper**

| **Antibody** | **Source** | **Identifiers** |
| --- | --- | --- |
| anti-beta Actin (Mouse monoclonal) | Servicebio | Cat#: GB12001 |
| anti-IL-1β (Rabbit polyclonal) | Bioss | Cat#: bs-6319R |
| anti-IL-6 (Rabbit polyclonal) | ABclonal | Cat#: A0286 |
| anti-cleaved-caspase 3  (Rabbit polyclonal) | Affinity | Cat#: AF7022 |
| anti-NINJ1 (Rabbit polyclonal) | Huabio | Cat#: HA500367 |
| anti-F4/80 (Rabbit polyclonal) | Servicebio | Cat#: GB113373 |
| Anti-CD68 (Rabbit polyclonal) | abcam | Cat#: ab303565 |
| anti-CD11b (Rabbit polyclonal) | Servicebio | Cat#: GB11058 |
| anti-CCR2 (Rabbit polyclonal) | Servicebio | Cat#: GB11326 |
| anti-CYP2E1 (Rabbit monoclonal) | Huabio | Cat#: ET1703-48 |
| anti-NRF2 (Rabbit polyclonal) | Huabio | Cat#: R1312-8 |
| anti-KEAP1 (Rabbit monoclonal) | Huabio | Cat#: HA721525 |
| anti-HO-1 (Rabbit monoclonal) | Huabio | Cat#: HA721854 |
| HRP conjugated anti-Mouse IgG  (Goat polyclonal) | Huabio | Cat#: HA1006 |
| HRP conjugated anti-Rabbit IgG  (Goat polyclonal) | Huabio | Cat#: HA1001 |
| Cy3 conjugated anti-Rabbit IgG  (Goat polyclonal) | Servicebio | Cat#: GB21303 |
| FITC conjugated anti-Rabbit IgG  (Goat polyclonal) | Servicebio | Cat#: GB22303 |

**Fig S1.** **Chronic alcohol consumption exacerbates APAP-induced oxidative stress and inflammatory response.** (a) The mRNA levels of oxidative stress-related genes in mouse liver tissues. (b) The mRNA levels of mitochondrial quality control-related genes in mouse liver tissues. (c) Representative immunofluorescence images of CCR2 and CD11b in mouse liver sections. The data were expressed as mean ± SEM and analyzed via two- way ANOVA. ns, no significant, *p < 0.05, **p < 0.01, ***p < 0.001.

**Fig S2. Macrophage depletion alleviates alcohol and APAP-induced oxidative stress.** (a) The mRNA levels of oxidative stress-related genes in mouse liver tissues. (b) The mRNA levels of mitochondrial quality control-related genes in mouse liver tissues. The data were expressed as mean ± SEM and analyzed via t-test. ns, no significant, *p < 0.05, ***p < 0.001.
